# Supplementary material for: Encapsulation of Nedaplatin in Novel PEGylated Liposomes Increases Its Cytotoxicity and Genotoxicity against A549 and U2OS Human Cancer Cells
Source: Pharmaceutics. 2020 Sep 10;12(9):863. doi: 10.3390/pharmaceutics12090863 (PMC7559812; doi:10.3390/pharmaceutics12090863)
Supplement: Supplementary file 1 [file pharmaceutics-12-00863-s001.pdf]

pharmaceutics

Supplementary Materials: Encapsulation of Nedaplatin in Novel PEGylated Liposomes Increases Its Cytotoxicity and Genotoxicity against A549 and U2OS Human Cancer

Salma El-Shafie, Sherif Ashraf Fahmy, Laila Ziko, Nada Elzahed, Tamer Shoeib and Andreas Kakarougkas

Materials and Methods

1. Analysis of ND by HPLC

Quantitative determination of nedaplatin was carried out using HPLC (Thermo Fischer Scientific DIONEX ultimate 3000 series), equipped with a photodiode array detector and utilising a BDS Hypersil® C18 reverse-phase column (250 mm × 4.6 mm, 5 mm). The mobile phase consisted of acetonitrile and deionized water (99:1) (v/v) at a flow rate of 1.2 ml/min, with the column temperature maintained at 40°C. The injection volume was 20 μL, and the effluent monitored at 210 nm. Nedaplatin concentration was determined using the constructed calibration curve (Figure S1).

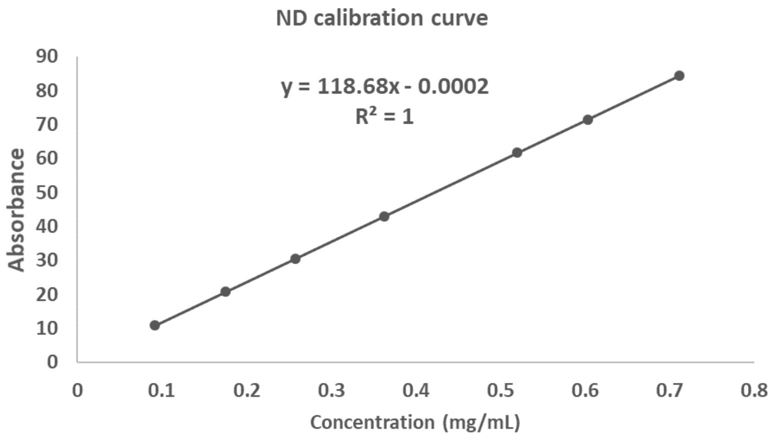

Figure S1. Calibration curve of ND showing a linear response in the range of 100-700 ng/ml with a correlation coefficient of 1.

2. Size statistics for liposomes

Table S1. Size statistics for non-loaded (void) liposomes.

Size d.nm	Intensity Percent	Size d.nm	Intensity Percent
0.4000	0.0	68.06	0.0
0.4632	0.0	78.82	0.0
0.5365	0.0	91.28	0.1
0.6213	0.0	105.7	7.6
0.7195	0.0	122.4	18.3
0.8332	0.0	141.8	25.3
0.9649	0.0	164.2	24.6
1.117	0.0	190.1	16.9
1.294	0.0	220.2	6.9
1.499	0.0	255.0	0.4
1.736	0.0	295.3	0.0
2.010	0.0	342.0	0.0
2.328	0.0	396.1	0.0
2.696	0.0	458.7	0.0
3.122	0.0	531.2	0.0
3.615	0.0	615.1	0.0
4.187	0.0	712.4	0.0
4.849	0.0	825.0	0.0
5.615	0.0	955.4	0.0
6.503	0.0	1106	0.0
7.531	0.0	1281	0.0
8.721	0.0	1484	0.0
10.10	0.0	1718	0.0
11.70	0.0	1990	0.0
13.54	0.0	2305	0.0
15.69	0.0	2669	0.0
18.17	0.0	3091	0.0
21.04	0.0	3580	0.0
24.36	0.0	4145	0.0
28.21	0.0	4801	0.0
32.67	0.0	5560	0.0
37.84	0.0	6439	0.0
43.82	0.0	7456	0.0
50.75	0.0	8635	0.0
58.77	0.0	1.000e4	0.0

Table S2. Size statistics for nedaplatin-loaded (LND) liposomes.

Size d.nm	Intensity Percent	Size d.nm	Intensity Percent
0.4000	0.0	68.06	0.0
0.4632	0.0	78.82	0.7
0.5365	0.0	91.28	5.0
0.6213	0.0	105.7	11.4
0.7195	0.0	122.4	17.2
0.8332	0.0	141.8	20.1
0.9649	0.0	164.2	19.0
1.117	0.0	190.1	14.6
1.294	0.0	220.2	8.6
1.499	0.0	255.0	3.2
1.736	0.0	295.3	0.3
2.010	0.0	342.0	0.0
2.328	0.0	396.1	0.0
2.696	0.0	458.7	0.0
3.122	0.0	531.2	0.0
3.615	0.0	615.1	0.0
4.187	0.0	712.4	0.0
4.849	0.0	825.0	0.0
5.615	0.0	955.4	0.0
6.503	0.0	1106	0.0
7.531	0.0	1281	0.0
8.721	0.0	1484	0.0
10.10	0.0	1718	0.0
11.70	0.0	1990	0.0
13.54	0.0	2305	0.0
15.69	0.0	2669	0.0
18.17	0.0	3091	0.0
21.04	0.0	3580	0.0
24.36	0.0	4145	0.0
28.21	0.0	4801	0.0
32.67	0.0	5560	0.0
37.84	0.0	6439	0.0
43.82	0.0	7456	0.0
50.75	0.0	8635	0.0
58.77	0.0	1.000e4	0.0

3. HEK293 MTT data

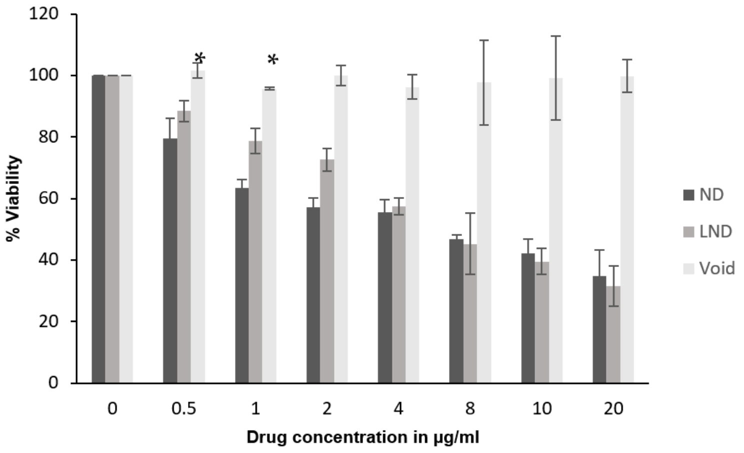

Figure S2. Evaluating cytotoxicity of free and liposomal nedaplatin after 72 hours of drug exposure using MTT assay in HEK293 cells.

4. Cytokinesis Block Proliferation Index

A549 and U2OS cells were seeded at a density of 1.5 x 10^5/well in 6-well plates containing sterilized glass coverslips. After adherence, media was replaced with drug-containing media at concentrations 0.1, 0.5 and 2 µg/ml of either ND or LND, or fresh medium for control wells. Cells were incubated with the drugs for 72 hours, but after 44 hours only of adding the drug, cytochalasin B was added to the drug-containing medium at a final concentration of 5 µg/ml, inhibiting cytokinesis from that point up to hour 72 within the presence of the drug. At 72 hours, cells were washed with PBS, fixed for 10 minutes in 4%formaldehyde, and nuclei were stained for 10 minutes using DAPI (5µg/ml) in PBS. Coverslips were then mounted on glass slides using fluoroshield-mounting medium and kept at 4ºC until further analysis.

One hundred cells per treatment were scored using an Olympus IX70 fluorescence microscope and categorized as mono- (M1), bi- (M2), or multi-nucleated (M3) cells. To quantify cytotoxicity, CBPI was calculated as: CBPI = M1 + 2(M2) +3(M3)/N, Where N is total number of counted cells (1-3). Experiments were carried out in biological triplicates for A549 and duplicates for U2OS.

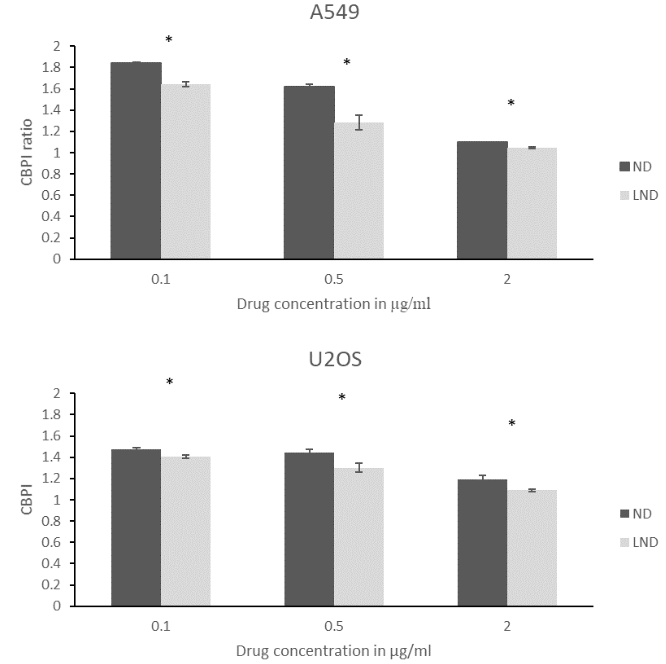

Figure S3. A dose-dependent decrease of CBPI values is noticed across increasing drug concentrations in both cell lines with A549 showing a steeper decrease and with LND yielding significantly lower CBPI values compared to ND ($p<0.05$).

5. Micronuclei formation assay with HEK293 cells

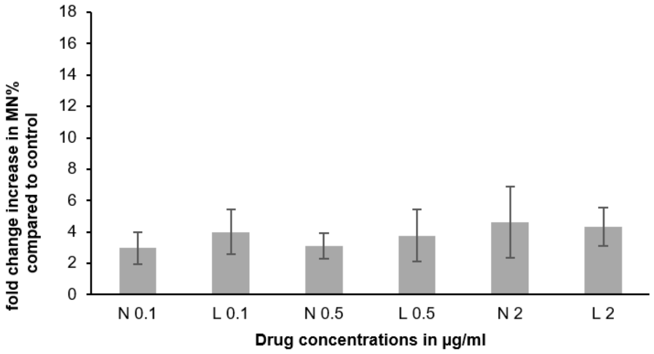

Figure S4. Genotoxicity of free and liposomal ND in HEK293 cells assessed through micronucleus formation induction. Fold-change in induction of micronuclei (MNi) across different concentrations compared to control MNi %.

6. γH2AX foci analysis with HEK293 cell

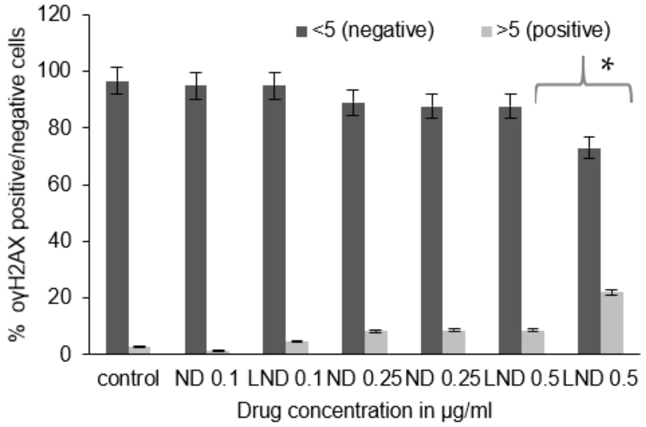

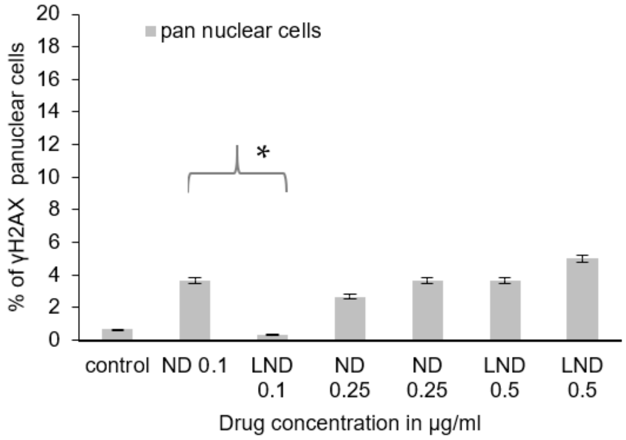

Figure S5. Quantification of γH2AX positive (top) and γH2AX panuclear (bottom) HEK293 cells treated with ND and LND. Multiple pair-wise t-tests showed the concentrations of treatment where a significant difference between LND and ND DNA damage is observed. These points are indicated by asterisks * in the charts above.

4. Apoptotic cell nuclear morphology

Cells in control cultures showed a vast majority of spherical, normal shaped DAPI-stained nuclei, with a percentage of cells shown undergoing mitosis. With increasing concentrations of ND or liposomal ND, nuclear morphological changes characteristic of apoptosis were observed. Apoptosis is characterized by chromatin condensation, DNA fragmentation and eventual disintegration into membrane-enclosed apoptotic bodies. The induction of apoptosis in A549 and U2OS cells by ND and LND liposomes was evaluated from chromatin condensation and apoptotic body formation, as well as earlier stage nuclear morphological changes similar to those reported in a study on nuclear morphological changes caused by Cisplatin in breast cancer cells at the early stages of apoptosis (4). These features include wrinkled and hyper-lobulated nuclear morphology. The percentage of apoptotic cells after treatment with ND and LND was not quantified, however, as adhesiveness of cells to substratum decreases at late stages of apoptosis, and cells were washed several times during the conducted experiments prior to fixation, and even past fixation, during permeabilization, loosely-attached cells such as apoptotic cells can be lost. However, with increasing drug concentrations, nuclear morphological changes were observed indicating execution of apoptosis as the cell-death program in response to this drug.

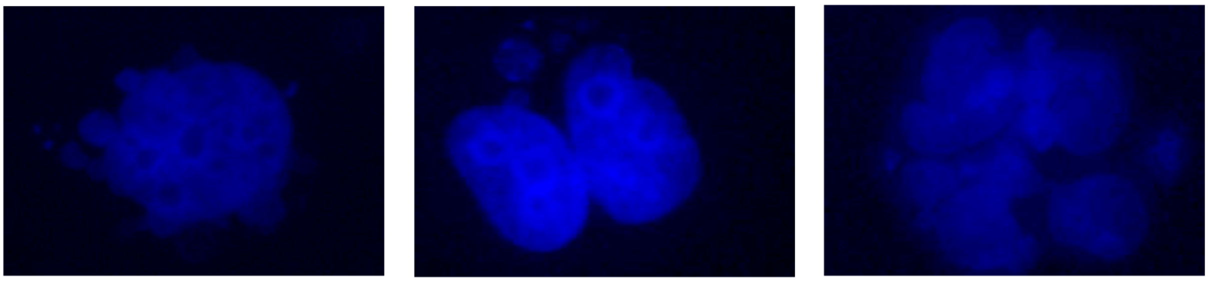

Figure S6. Apoptotic cell nuclear morphology in A549 following treatments for 72 hours with ND or LND.

5. Micronuclei formation assay

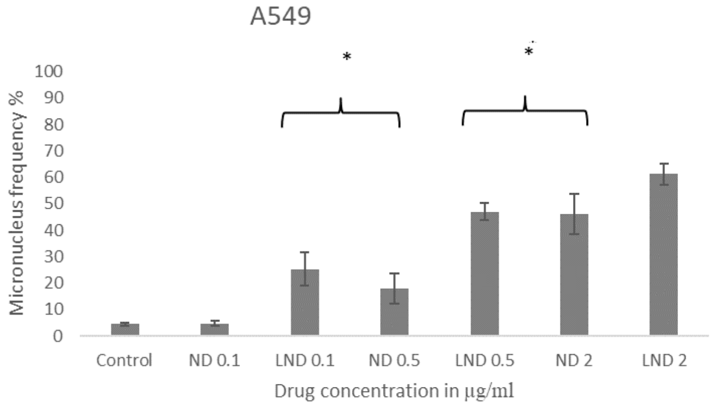

(A)

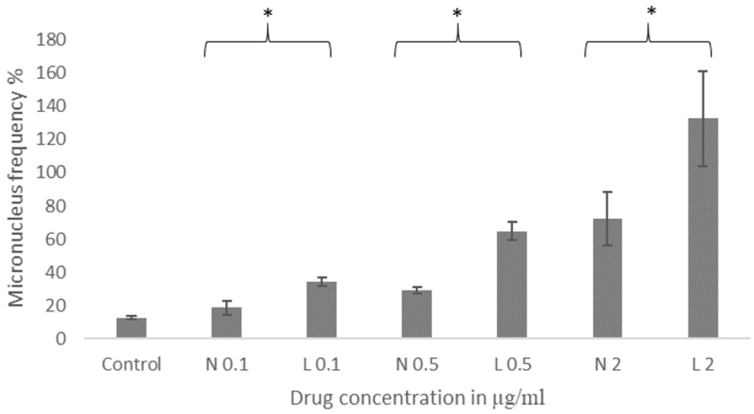

(B)

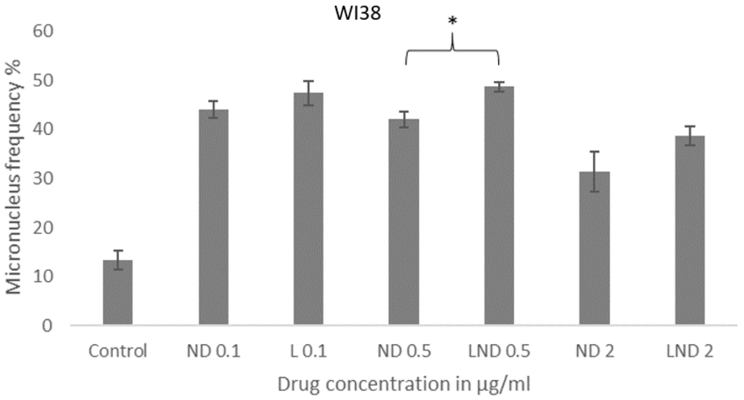

(C)

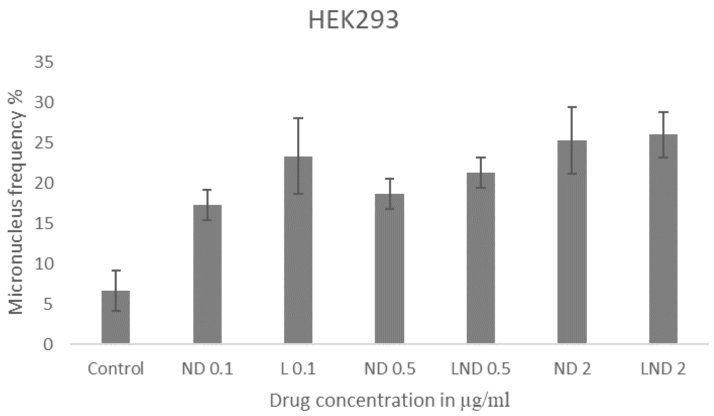

(D)

Figure S7. Genotoxicity of free and liposomal ND in different cell lines assessed through micronucleus formation induction. (A) A549; (B) U2OS, (C) WI-38 and (D) HEK293 showing Micronucleus frequency per 100 binucleated cells across different drug concentrations. An overall statistically significant increase in MNi formation was observed with LND compared to ND (p-value<0.05) in cancer cell lines A and B, but not in normal cell lines C and D. Multiple pair-wise t-tests show the concentrations at which a significant difference between both drugs was observed. These points are indicated by asterisks (*).

References

1. Fenech M. Cytokinesis-block micronucleus cytome assay. Nat Protoc. 2007;2(5):1084–104.
2. Kalweit S, Utesch D, Hude von der W, Madle S. Chemically induced micronucleus formation in V79 cells--comparison of three different test approaches. Mutat Res. 1999 Feb 19;439(2):183–90.
3. Kirsch-Volders M, Sofuni T, Aardema M, Albertini S, Eastmond D, Fenech M, et al. Report from the in vitro micronucleus assay working group. Mutat Res. 2003 Oct 7;540(2):153–63.
4. Krajcí D, Mares V, Lisá V, Spanová A, Vorlícek J. Ultrastructure of nuclei of cisplatin-treated C6 glioma cells undergoing apoptosis. Eur J Cell Biol. 2000 May;79(5):365–76.
